# Assessment of Risk and Resilience of Terrestrial Ecosystem Productivity under the Influence of Extreme Climatic Conditions over India

**DOI:** 10.1038/s41598-019-55067-0

**Published:** 2019-12-12

**Authors:** Srinidhi Jha, Jew Das, Manish Kumar Goyal

**Affiliations:** Discipline of Civil Engineering, Indian Institute of Technology, Indore, 453552 India

**Keywords:** Climate sciences, Hydrology

## Abstract

Analysing the link between terrestrial ecosystem productivity (i.e., Net Primary Productivity: NPP) and extreme climate conditions is vital in the context of increasing threats due to climate change. To reveal the impact of changing extreme conditions on NPP, a copula-based probabilistic model was developed, and the study was carried out over 25 river basins and 10 vegetation types of India. Further, the resiliency of the terrestrial ecosystems to sustain the extreme disturbances was evaluated at annual scale, monsoon, and non-monsoon seasons. The results showed, 15 out of 25 river basins were at high risks, and terrestrial ecosystems in only 5 river basins were resilient to extreme climatic conditions. Moreover, at least 50% area under 4 out of 10 vegetation cover types was found to be facing high chances of a drastic reduction in NPP, and 8 out of 10 vegetation cover types were non-resilient with the changing extreme climate conditions.

## Introduction

Net primary productivity (NPP), a key indicator of ecosystem functioning, is the rate at which energy is transferred as biomass by autotrophs to the consumers in the terrestrial ecosystems. Change in climatic conditions directly influence the NPP occurrence and distribution^1^. Analysing the response of NPP to change in the extreme climatic conditions provides an insight into the risk and resiliency of the terrestrial ecosystem^2^. For instance, Grimm *et al*.^3^ found that change in climatic conditions results in biome shifts, forest growth and mortality, and ecosystem state changes which in turn contribute to the alteration in NPP. Sharma and Goyal^4^ assessed the status of ecosystem resilience to climatic perturbations and reported that the response of NPP to change in climatic conditions is also a function of different vegetation types. Pan *et al*.^5^ found that variation in the NPP in different regions of the world is attributed to variability in precipitation, temperature and several climatic factors. In a recent study, it was found that quantification of spatio-temporal variability in NPP is essential in determining how the ecosystem will respond to future changes in climate and land use^6^. Conventional analysis of the climate controlling factors on terrestrial ecosystem productivity involves the investigations based on the long-term variability in mean temperature and precipitation and their relationship with NPP. In relation to climate-ecosystem interactions, ecosystem functioning indicators such as NDVI,NPP and CO_2_ fluxes at times have been directly compared to climate indices^7–9^. A number of NDVI-derived indices such as Vegetation Condition Index (VCI), Standardized Vegetation Index (SVI) and Vegetation Drought Response Index (VegDRI) have also been used to understand impact of extreme climate on terrestrial ecosystems^10,11^. These indices, although provide a robust description of ecosystem state, are often insufficient in understanding the mechanism of risks generated due to climatic condition^11^. Also, the interaction of climate-soil-plant systems for energy and material exchange is complex and involves many hydrological and biogeochemical processes^12^. The dependence of NPP and mean annual precipitation has been widely studied in the past^13,14^. A study by Hoeppner and Dukes^15^ indicates that the NPP is significantly related to the global temperature anomalies and there is substantial evidence of the linkage between NPP variability and temperature changes across different ecosystems in multiple climate zones^16–18^. Miranda *et al*.^19^ advocated that lower water availability significantly alters the vegetation cover and its productivity. A recent study by Zhao *et al*.^20^ indicates that that water-carbon cycle is closely  coupled and water availability plays a crucial role in determining the distribution of NPP in a region. Sinha *et al*.^21^ pointed out that the water balance of a catchment is influenced by change in vegetation and remains intricately coupled with several other regional characteristics. Water availability specially for the rainfed agroecosystems need to be properly understood and managed to meet food security and climate change problems^22^. Also, the global climate change and human intervention could influence the water-carbon coupling process and need to be explored for a better understanding of sustainable ecosystem management^23,24^.

Conventionally, studying the ecosystem productivity involved experimental investigation of the response of ecosystem variables (such as plant diversity, vegetation cover, nutrient concentration) as well as statistical or empirical-based analysis of ecosystem productivity to the extreme climatic conditions^19,25–28^. The existing experimental approaches have limited applicability as it is challenging to incorporate the wide spatio-temporal variability in ecosystem functioning. Currently, modeling the response of the ecosystem to climate change is mostly based on assessing the impact of climatic trends such as changes in precipitating, temperature warming and increase in *C* concentration. In addition, most of the conventional statistical and empirical approaches are formulated based on the assumption of stationarity, scale invariance, etc., which are not advisable in the context of climate change studies^29,30^. The knowledge about specific impacts of climate extremes such as severe drought, prolonged precipitation deficit and extreme temperature on terrestrial ecosystem has become critical to society and science. These extreme events are spatio-temporally varied due to several factors such as intensified hydrological cycles, global influences like climatic oscillations and regional characteristics such as land use, topography etc^31–33^. Since the response of terrestrial ecosystems to extreme climatic events is varied and complex, there has been limited research in this area. Modeling the behaviour of ecosystems during and after climate extremes at larger spatial scales and over longer periods requires more in‐depth knowledge on possible response mechanisms at different scales (such as biome types, river basin scales, climate types etc.). Therefore, more efficient techniques are required to understand the association between the dynamics of ecosystem’s response to extreme climatic conditions and its implication to the society.

In general, the impact of extreme climate events on terrestrial ecosystem have been examined by univariate analysis of climate variables^34,35^. Since, the significant nonlinear correlation between climate variables and ecosystem functioning indicator might not be captured during univariate analysis, many researchers have recommended the use of joint distribution to describe the characteristics of such events^36–38^. Given the fact that ecosystem-climatic interaction is complex, modelling the possible influence of extreme climate on ecosystem productivity from joint likelihood point of view is more suitable. Considering this we performed a novel and comprehensive multivariate copula based probabilistic study for examining the relationship of NPP and with different climate variables over all major river basins and land cover types of India.

Copulas have been widely utilized in a variety of fields such as finance^39,40^, hydrology^41,42^, signal processing^43,44^, medical^45,46^, climate studies^47,48^. However, the application of copulas in assessing the dependence of ecosystem productivity on climatic conditions is rare. In this study, we employ a probabilistic approach using copula framework to investigate the likelihood of changes in ecosystem productivity under the influence of extreme climate conditions. The ability to recover from the impacts of extreme climatic conditions is also important in context of productivity. To incorporate this aspect, we have further estimated the resilience of terrestrial ecosystems across India. To the best of author’s knowledge, present investigation is the first to assess the dependence of ecosystem productivity with extreme climatic conditions through a probabilistic approach considering multiple climate drivers over India. The study provides the estimates of risks to the terrestrial ecosystems in all major river basins and land cover types in the country incorporating the possible extreme behaviour of precipitation, temperature and soil moisture content. Moreover, we provide a high-resolution map of resilience of the terrestrial ecosystems to dry conditions across India. The study is a novel attempt to identify the possible mechanism behind the high risk and low resilience of terrestrial ecosystems such that plans and policies for sustainable terrestrial ecosystem management could be framed. In light of this, the objectives of the work are (i) to estimate the likelihood of drastic deterioration in ecosystem productivity when subjected to extreme changes in climatic conditions; (ii) to investigate the vulnerability of terrestrial ecosystems across the country at different temporal (i.e. monsoon, non-monsoon seasons and annual time period) and spatial scales (i.e. different vegetation types and river basins); (iii) to identify the most dominant climatic factor in augmenting damage to ecosystem productivity and; (iv) to assess the resiliency of terrestrial ecosystems to the hydro-climatic disturbances.

## Results

**Figure 1:**
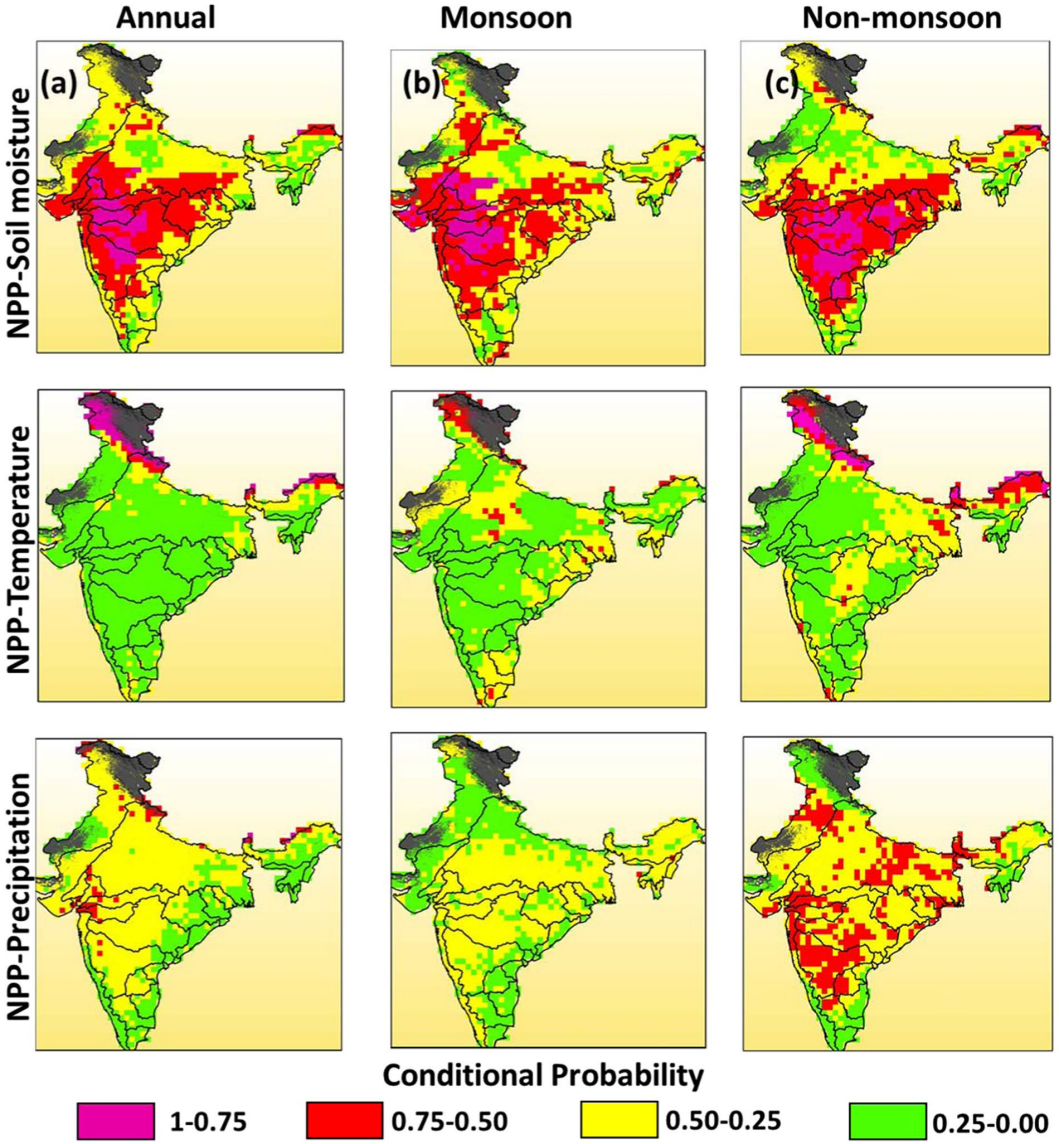
Conditional probabilities of severe damage to ecosystem productivity (NPP ≤30%) on (**a**) annual scale, and in (**b**) monsoon and (**c**) non-monsoon seasons in highly stressed scenarios (n ≤20%) of soil moisture, temperature and precipitation (top to bottom).

We set a threshold of NPP value corresponding to 30^th^ percentile $({n}_{NPP}\le 30 \% )$ as an indicator of the drastic reduction in ecosystem productivity at a point. The conditional probability distribution corresponding to this NPP threshold was estimated in stressed climate scenarios of precipitation, soil moisture content and temperature, respectively. The stressed climatic scenario is defined by selecting the 20^th^ percentile $(n\le 20 \% )$ of precipitation, soil moisture content and temperature. A copula-based bivariate probabilistic model was developed to model the joint behaviour of NPP and climate variables and eventually estimate the conditional likelihood of extremely low NPP (${n}_{NPP}\le 30 \%$). The analysis was performed for 25 major river basins and 10 major land cover types of India in two seasons (monsoon and non-monsoon) and annual scale (See Figure S1 and Table S1 in the supplementary information for river basin and land cover nomenclature). Further, please refer to the Data and Methodology section for details about the data used and the probabilistic model employed for the analysis. The obtained likelihood of $({n}_{NPP}\le 30 \% )$ was divided into four classes of extreme (1.00-0.75), high (0.75-0.50), moderate (0.50-0.25) and low (0.25-0.00) risks. The investigation at annual time scale revealed that majority of river basins were falling into high-risk category when subjected to very low soil moisture values (Fig. 1a). Lowering the temperature was least disturbing to the ecosystem productivity except in high-altitude cold regions. The high risks due to stressed temperature at annual scale were observed only in 4 out of 25 river basins which collectively add up to less than 10% of the country’s area (Table 1). However, reducing the precipitation induces moderate risks of serious damage (i.e., NPP ≤30^*th*^ percentile) to productivity in most of the river basins. Small areas of the Western Ghats, river basins in the eastern coast such as FRMGP, EFRGKB and EFRPCB and northeast region namely, Barak and Brahmaputra were prone to high risks. Productivity in the river basins such as Mahi and Tapi was most likely to be hit by extreme changes in the annual precipitation as 22.11% and 21.05% of their area exhibited high risks, respectively. More importantly, the analysis of annual NPP and soil moisture levels reveals that 100% area of three river basins (Mahi, Sabarmati and Tapi) were prone to high risks. However, the river basins on the east coast and the northeast region were relatively safer. Area-wise, the most susceptible river basins were Godavari and Ganga of which, a total area of 476798.5 km^2^ came under the high-risk zone. India receives most of its rainfall in the monsoon season. Similar to results at the annual scale, stressed temperature scenario was not a major threat in monsoon season, and only about 6.5% of country’s area was at high risk (Table S2 and Fig. 1). However, it was observed that extreme reduction in soil moisture even in the monsoon season might cause severe damage to the ecosystem productivity of about 38% area of the country. More than 50% area of 8 out of 25 river basins was prone to severe damage due to lower soil moisture levels. In addition, reducing the precipitation, soil moisture and temperature to the same level (i.e. n ≤ 20%) more significantly damage productivity in the non-monsoon season. The temperature in non-monsoon months may bring more substantial risks to the ecosystem productivity in high-altitude basins as compared to the annual scale and monsoon season (Table S1). It can be observed that about 61.54% area of the Brahmaputra basin was under high risk followed by the Indus river basin’s 28.18%. Further, ecosystem productivity in about 24% area of the country was at high risks in lowered precipitation scenario in the non-monsoon season as compared to 1% in the monsoon season. Productivity in Krishna river basin was found to be most vulnerable as 68.17% of its area was at high risks in the non-monsoon season.

**Table 1:** Area susceptible to witness damage to ecosystem productivity at annual scale in stressed climate scenario (n ≤ 20%) for different river basins obtained from different sets of NPP and climate variables.

Id	Basin	NPP-Soil Moisture		NPP-Precipitation		NPP-Temperature	
		Area (%)	Area (*km*^2^)	Area (%)	Area (*km*^2^)	Area (%)	Area (*km*^2^)
1	Indus	2.89	13244.41	5.924855	27151.04	44.80	205288.38
2	Ganga	29.46	235088.31	4.232365	33773.25	5.98	47679.88
3	Brahmaputra	13.29	25164.38	6.643357	12582.19	21.33	40395.46
4	Barak	0.00	0.00	0	0.00	0.00	0.00
5	Godavari	85.28	241710.51	0.700935	1986.66	0.00	0.00
6	Krishna	80.56	189395.09	2.253521	5297.76	0.00	0.00
7	Cauveri	20.34	15893.29	0	0.00	0.00	0.00
8	Subarnarekha	25.00	5959.99	0	0.00	0.00	0.00
9	BB	28.95	14568.85	0	0.00	0.00	0.00
10	Mahanadi	76.44	96684.20	0	0.00	0.00	0.00
11	Pennar	44.29	20528.84	0	0.00	0.00	0.00
12	Mahi	100.00	37746.57	21.05263	7946.65	0.00	0.00
13	Sabarmati	100.00	28475.48	2.325581	662.22	0.00	0.00
14	Narmada	97.71	84764.23	13.74046	11919.97	0.00	0.00
15	Tapi	100.00	62910.96	22.10526	13906.63	0.00	0.00
16	EFRMGB	1.41	662.22	0	0.00	0.00	0.00
17	EFRGKB	0.00	0.00	0	0.00	0.00	0.00
18	EFRKPB	0.00	0.00	0	0.00	0.00	0.00
19	EFRPCB	0.00	0.00	0	0.00	0.00	0.00
20	EFRSCB	0.00	0.00	0	0.00	0.00	0.00
21	Luni	46.59	86088.68	2.150538	3973.32	0.00	0.00
22	MRBB	0.00	0.00	0	0.00	0.00	0.00
23	MRMB	0.00	0.00	0	0.00	0.00	0.00
24	ANLIB	0.00	0.00	0	0.00	52.50	13906.63
25	WG	37.42	38408.79	2.580645	2648.88	0.00	0.00

Table S3 shows the areas falling under the high-risk class at different vegetation type levels in the monsoon, non-monsoon season and annual scale. Stressed temperature scenario induces such high risks in 57.14% of Evergreen Needleleaf Forests at annual scale. Further, ecosystem productivity of 45.30% of the Croplands was at high risks in lower temperature scenario. It was found that the Evergreen Needleleaf Forests were most sensitive to the extreme climate conditions as 28.57% and 35.71% of their areas was prone to touch the 30^th^ percentile threshold in stressed soil moisture and precipitation condition, respectively. Reduced annual soil moisture levels were highly threatening to the productivity of about half (49.61%) of the Croplands. This threat to the Croplands was more intensified in the monsoon season, and 54.58% of its area fell under high-risk zones. However, extremely stressed precipitation could induce high risks only in small portion of Deciduous Needleleaf Forests (3.12%) and Deciduous Broadleaf Forests (3.52%). However, lower temperature remained a threat to Evergreen Needleleaf Forest even in monsoon season. The non-monsoon season, as discussed, was found to be most unfavourable time period for the smooth functioning of terrestrial ecosystems in the country. An enormous 85.71% of the Evergreen Needleleaf Forests was found to be at high risk in lower temperature scenario. Furthermore, lower soil moisture content caused maximum amount of risks in the Woody Savannas (65.89%), followed by Deciduous Broadleaf Forests (48.15%), Croplands (43.83%), Croplands/Natural Vegetation Mosaic (42.94%), Savannas (38.20%), Grasslands (35.36%), Mixed Forests (29.08%), Deciduous Needleleaf Forests (21.88%) and Evergreen Broadleaf Forests (5.23%). Stressed precipitation in the non-monsoon season was found to be affecting Croplands the most (36.83%).

The resilience of terrestrial ecosystems to recover from the damage to their productivity was calculated and related to different river basins and vegetation cover types. At annual scale, Tapi river basin (71.58%) was found to be most non-resilient followed by Krishna (71.55%) and EFRKPB basin (63.89%) (Table S4). Similarly, the most resilient river basins were EFRGKB (7.69%), EFRMGB (14.08%), BB (19.74%), ANLIB (22.5%) and EFRSCB (24.44%). At annual scale, at least one-third area of 19 out of 25 river basins was found to be highly non-resilient (Figure S2 and S3). Whereas, the analysis of dry conditions in the monsoon months led to the conclusion that at least one-third area of 17 out of 25 river basins was highly non-resilient. Opposite to the result we observed at the annual scale, a dry event in the monsoon season led to more long-lasting impact to the ecosystem productivity in the EFRGKB basin turning 84.62% of its area non-resilient. The most resilient basins in monsoon seasons belonged to the high rainfall regions such as the northeast (MRMF and MRFB), east coast (EFRSC and EFRPCB) and the Western Ghats. Less than 20% of these river basin’s area were non-resilient to a dry condition in monsoon months. Moreover, a dry condition in the non-monsoon season was found to be most disturbing to the EFRGKB basin as 92.31% of its area was found to be non-resilient. At least half area of 16 out of 25 river basins was non-resilient in a dry non-monsoon month. The resilience was also related to different biome types, and it was observed that Evergreen Needleleaf Forest was most susceptible to damage at the annual scale. Ecosystem productivity in about two-thirds (64.29%) of its area was not able to recover from the effect of a dry condition (Table S5). A significant area (43.31% or 664207.24 km^2^) of the Croplands was found to be non-resilient to the dry condition at the annual scale. In the monsoon season, 57.21% of the Croplands was found to be non-resilient which points out the vulnerability of Indian agriculture lands. The Croplands were most incapable of facing a dry condition followed by Woody Savannas (51.94%), Savannas (49.44%), Grasslands (48.62%), Deciduous Broadleaf Forests (48.15%), Evergreen Needleleaf Forests (42.86%), Croplands/Natural Vegetation Mosaic (42.34%), Mixed Forests (41.13%), Deciduous Needleleaf Forests (40.63%) and Evergreen Broadleaf Forests (28.76%). Similar to the monsoon months, a dry condition in the relatively drier non-monsoon season was observed to cause more damage to the Croplands of the country. It was found that ecosystem productivity in 61.31% (940353.22 km^2^) of its area was not able to regain its original state in the driest month during 2001–2010. Most resilient forest cover was Deciduous Broadleaf Forest about two-thirds (65.62%) of which was resilient to dry condition in non-monsoon season.

## Discussions

**Figure 2:**
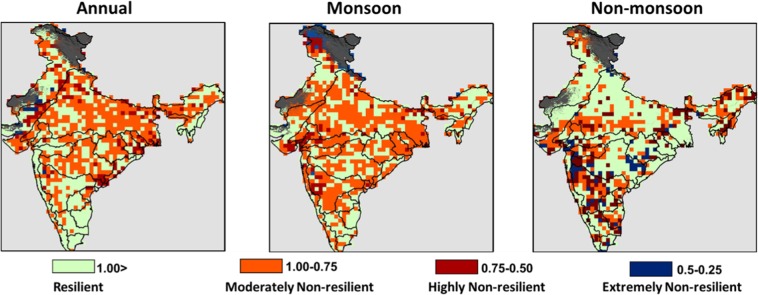
Distribution of resilience (Ri) values at annual scale, and in monsoon and non-monsoon seasons.

This study was aimed at investigating the possible changes in ecosystem productivity under the influence of extreme climatic conditions. The preliminary correlation analysis of the data suggested that terrestrial ecosystem productivity is significantly and highly influenced by the climatic conditions. However, this dependence is complex and required an efficient framework for estimating the risk and resilience of terrestrial ecosystems^18,49^.We analysed the likelihood of NPP dropping down to the 30^th^ percentile threshold in stressed scenarios of precipitation, soil moisture content and temperature using multivariate probabilistic approach in different seasons and annual scale. The results agree with previous studies that variations in the terrestrial ecosystem productivity is strongly governed by change in climatic conditions^50–53^. It was found that stressed soil moisture levels were the most crucial in governing the ecosystem productivity in many regions. This conclusion is in accordance with the previous findings indicating soil moisture strongly dominates the productivity of terrestrial ecosystems^54–56^. Productivity in the non-monsoon season was most susceptible to damage under extreme conditions. This is logical, as the non-monsoon season is also the drought prone duration in the country where limited water availability limits the productivity of plants^57^. In stressed soil moisture conditions, the river basins in the peninsular as well as the north-western area were highly vulnerable. The high risk in Luni, Mahi and Pennar river basins is due to the fact that these are one of the most drought-prone basins of the country^58–60^. The threat to productivity in these regions directs towards the danger to vegetation growth and activity. The non-monsoon season also comprises of months in which temperature is at its lowest value which makes the season most unfavourable for ecosystems in high-altitude zones. Kusre and Lalringliana^61^ have shown that winter droughts in the Himalayan region of India is major cause of concern calling for urgent management measures in the region. It was observed that lowering the temperature had least impact on the NPP in other areas and the productivity was affected only in some high-altitude river basins. This result asserts that NPP has least coherence with temperature in India^62^. The rate of biomass yield is a product of the growth duration and mass accumulation which is primarily influenced by the amount of sunlight intercepted by plants over an optimum range of temperature^63^. It is well known from very early studies that low light conditions may severely affect the primary productivity^64,65^. Therefore, extreme or high risks in these regions during lower temperature are reasonable. The effect of highly stressed precipitation was less severe as compared to stressed soil moisture content. For example, 96.96% of Godavari river basin’s area was under high risk in lowered soil moisture condition; however, only 33.64% of the basin’s area was vulnerable in lowered precipitation condition (Table S1). Research show that Godavari river basin experience very high rainfall and temperature variability, hence, is susceptible to risks due to changing climatic conditions^66,67^. The tendency of soil moisture being more dominant factor than precipitation in causing extreme damage to the ecosystem productivity was observed in 18 out of 25 river basins. This indicates that most of the river basins in India lack the mechanism to hold incident precipitation and support the productivity. It has been pointed out that preserving the rainfall is the most crucial aspect of sustainable ecosystem management^68,69^. Further, there are several studies available which support the idea that precipitation and soil moisture are connected through a number of feedback processes and alteration in one affects the occurrence and distribution of other^70,71^. From the analysis, it can be observed that lack of precipitation in India lowers the soil moisture content to dangerously low levels which in turn damages the ecosystem productivity. The results are in line with other studies conducted for India which suggest that water availability is primary driver in alteration of NPP^62,72^. Similar behaviour was observed in the monsoon season (Table S2). Extremely low precipitation was not the main factor behind possible reduction in primary productivity in the monsoon months. The water scarcity resulted in the inadequate soil moisture availability for plant’s growth lead to high risks in more than 50% area of 8 out of 25 river basins. When considered the annual time scale, extremely stressed precipitation resulted in high risks in only small areas of some river basins (Table 1). High risks of severe reduction in ecosystem productivity in more than 20% area were observed only in two river basins (Mahi and Tapi). However, in case of lower soil moisture, whole Mahi and Tapi river basins were experiencing high threats. This is in accordance with the results from other studies which have pointed out that western parts of the country are highly sensitive to climatic conditions^73,74^. Further, the investigation facilitates the understanding of seasonal as well as temporal variation in the functioning of terrestrial ecosystems of India. The response to the extreme condition is also a function of different biome types^75,76^. We analysed the likelihood of ecosystem productivity dropping to 30^th^ percentile in extreme conditions and found that each vegetation type has a unique response to stressed climatic conditions. In the non-monsoon season, Evergreen Needleleaf Forests were found to be most sensitive to the drop in the temperature (Table S1). Previous works show that temperature is a significant driver of variability in NPP of evergreen forests^77,78^. We found that 85.71% of these forest types were highly sensitive to extreme temperature change which is alarming for its existing flora and fauna. However, these forests types were completely safe from high risks in extremely low scenarios of both precipitation and soil moisture content. Further, in both the seasonal and annual time scales, a large extent of Indian Croplands was prone to severe damage in its productivity. This threat to Indian Croplands has been pointed out by previous works which validates the conclusions of our findings^79,80^. Further, studies recommend that a prolonged water deficit condition can lead to strong decline in NPP, particularly for India where precipitation has been found to be governing more than 60% of variations in the NPP^5,81^. This sensitivity in productivity is concerning for the food security of India’s huge population. The Ganga river basin is home to more than 600 million people and more than 40% of India’s Gross Domestic Product (GDP) is generated in this region^82^. We found that more than one-third of its area was facing high risks due to lower soil moisture content (Table 1). Therefore, the policymakers urgently need to look for possible ways to preserve the incident precipitation in the river basin in order to ensure enough soil moisture availability during the cropping season. The extreme conditions may also affect the production of major crops of the country such as Cotton, Wheat, Millet and Pulses as most of the central and peninsular India were susceptible to extreme or high risks and low resilience (Fig. 1 and Fig. 2). Although, the Croplands were highly threatened under lower soil moisture and precipitation scenarios, low temperature had negligible impact on its productivity. Low temperature in these regions can be associated with less evapotranspiration hence, greater soil moisture availability which may favour the growth of crops.

Once the likelihood of severe reduction in ecosystem productivity was estimated, we analysed the resilience to dry conditions. The productivity of the ecosystem is governed by a variety of processes. Particularly for India, the association of vegetation productivity with climate is highly influenced by seasonal variability^83^. Hence, to investigate the influence of seasons on recovering capacity we computed the resilience in all time scales. First, the driest period during 2001–2010 was identified and then primary productivity at a grid point during the same time period was compared with the temporal mean primary productivity. The resilience of terrestrial ecosystems for a climate vulnerable country like India was expected to be low. The analyses suggested that more than 58% of India’s terrestrial ecosystems were highly non-resilient to dry condition. Earlier studies in different regions of the country have highlighted the poor recovering capacity after any extreme climatic event^84,85^. Most of the river basins were unable to recover from the impact of a dry event and regain its mean productivity. At least 70% area of basins in the arid regions of the country such as Mahi, Tapi, and Sabarmati was highly non-resilient which indicates that damage induced by an extreme condition may persist for a longer period (Table S4 and Figure S3). More importantly, the river basins which receive high rainfall were also found to be poorly capable of maintaining its productivity as more than half area of Barak, Brahmaputra and Western Ghats were non-resilient. The results from copula analysis suggested that chances of extreme changes in productivity were generally moderate under lower soil moisture and precipitation (Fig. 1). However, the poor resilience to dry condition indicates that even these river basins were not completely safe from risks (Fig. 2). In addition, more than 500000 km^2^ (65%) of the Ganga river basin was non-resilient which adds to the concern as more than 33% of its area showed high likelihood of NPP dropping to 30^th^ percentile threshold (Figure S2). Therefore, it can be concluded that not only the chances of reduction in productivity is high, but also, the river basin might not sustain the risk subsequently damaging its crop yields. Extremely or highly resilient river basins were majorly located in the north-western or southern part of the country, and most resilient river basins were located in north-east India (Fig. 2). Studies suggest that changing climate conditions will have serious implications for Indian forests^78,79^. We analysed the resilience with respect to different forest types and found that it was strongly related to both the forest type and time scale. We found that more than half of every forest type was non-resilient in one time scale or the other (Table S5). The vegetation cover types with half of their area showing high risk and resilience were Evergreen Needleleaf Forests, Woody Savannas and the Croplands. Individually, more than half area of 8 out of 10 vegetation types was non-resilient.

Our study facilitates the understanding of the impact of extreme climatic conditions on terrestrial ecosystem functioning across India. The analysis was performed to identify the sensitive regions and time during the year in which the maximum threat to ecosystem productivity may occur. The study on river basins and land cover scales provides in-depth identification and characterization of the risk factors and delivers crucial inputs for terrestrial ecosystem management and sustainable policymaking. The study put forward all-India assessment of regional impact of global climate change over ecosystem functioning across different river basins and land cover types. The results serve to the mapping of climatic factors influencing the ecosystem productivity at multi-spatial and temporal scale which is necessary to understand the complex response of terrestrial ecosystems to climate change. The framework will help in developing coping strategies for the adapted management of these ecosystems.

## Data and Methodology

### Considered vegetation types and river basins

To understand the influence of spatio-temporal variability on ecosystem productivity we selected 25 river basins across India as classified by India-WRIS, (2014)^86^. Please refer to Figure S1 for basin IDs and Table S1 in the supplementary information for the river basin nomenclature. Vegetation distribution is also one of the primary factors which governs the ecosystem productivity. Moreover, India’s vegetation significantly changes at different temporal and spatial scales. To investigate the ecosystem dynamics in the context of vegetation types, we identified 10 major land covers from the of 5.1 MCD12Q1 data of Land Cover Institute (LCI, https://landcover.usgs.gov/global_climatology.php)^87^. Please see Figure S1 for vegetation types, their codes and spatial distribution.

### Climate data and net primary productivity

We analysed the effect of extremely low mean monthly precipitation, soil moisture content, and temperature for the decade 2001–2010 on the NPP over different river basins and vegetation types in India. The gridded precipitation data (0.25° × 0.25°) was extracted from a high spatial resolution IMD4 (India Meteorological Department 4) data set^88^ which has been prepared from the records of daily rainfall from a dense network of 6955 rain gauge stations in the country. The data set has been prepared after making quality control of basic rain-gauge stations. It was found that IMD4 data is comparable with existing gridded daily rainfall data sets such as IMD1, IMD2, IMD3 and APHRO. IMD4 data very efficiently captures the spatial distribution of rainfall in high rainfall regions of India such as the West Ghats and north-east India. Because of its effectiveness in capturing the spatio-temporal variability of Indian monsoon IMD4 has been used in many climate change studies^89–91^. The temperature data prepared using Shepard’s technique employed on 395 observational stations in the country was also sourced from IMD. The error related to these data sets has been calculated and found to be less than 0.5 °C^92^. This data set was also verified and compared with other available high-resolution data sets. It was found that the data is comparable and very well describes the magnitude and frequency of temperature indices such as heat waves, cold waves temperature anomalies. Several researchers have employed this data made available through National Data Centre, Pune in a variety of applications^93–95^. Similarly, the soil moisture values were extracted from the widely used Climate Prediction Centre (CPC) data sets prepared by the Earth System Research Laboratory of National Oceanic Atmospheric Administration (ESRL-NOAA) (http://www.esrl.noaa.gov/psd/data/gridded/data.cpcsoil.html). The CPC soil moisture data is prepared from a bucket water balance model using global precipitation at 17,000 gauge stations worldwide and temperature from the global Reanalysis. The validation of data at annual as well as inter-annual levels proves the it efficiently captures the temporal and spatial variability. Some of the works which used this data in different parts of the world show that CPC soil moisture data is reliable and can be engaged in understanding the ecosystem climate interaction^96–98^. We obtained the global annual NPP values from the NASA Earth Observation System (EOS) program’s MOD17A2 datasets (https://neo.sci.gsfc.nasa.gov/view.php?datasetId=MOD17A2_M_PSN). The MODIS net primary productivity data set is available from 2000 at 8-day, monthly and annual time steps. The data has been prepared by MODIS land science team after performing a number of quality assurance, calibration and validation activities. The data has been compared with observations from various validation sites, operational scientific network data as well as the data derived from high resolution satellites (e.g., ASTER, AVHHR, MISR, TM/ETM+) or airborne surveys. In addition, the comparison with similar data products derived from different sensor is also done to check the spatial and temporal consistency between products. Readers are advised to refer Running *et al*.^99^ to understand the preliminary plan for MODIS data integration into terrestrial ecosystem monitoring. In addition, several works utilizing this validation strategy following the MODIS launch can also be referred^99–102^. These studies show that the validation has been performed by using data sets well distributed over time and space which makes it reliable for use in our study. The application of MODIS data in previous studies have proved its efficiency in representing terrestrial ecosystem state. For instance, Nayak and Dadhwal^103^ in a significant study, checked the consistency of NPP estimates from CASA model (Carnegie–Ames–Stanford Approach), field-based observations, and MODIS data. It was found that the CASA-based annual NPP, NPP from MODIS data and ground-based NPP were in good agreements with each-other. Huang *et al*.^104^ studied the dynamics of drought (using SPEI) and its impact over terrestrial net primary production at global level implying that MODIS data was efficient in depicting the state of terrestrial ecosystems. Huang *et al*.^105^ and Xia *et al*.^106^ explored the relationship between carbon and water cycle to understand the impact of climate change on terrestrial ecosystem productivity using MODIS NPP product to infer significant results. Bastos *et al*.^107^ and Poulter *et al*.^108^ investigated the possible reasons behind the highest annual NPP growth recorded in 2011 and observed that high rainfall in arid regions was the main cause behind such anomaly. Cleveland *et al*.^109^ examined the role of NPP in governing the mechanism of biogeochemical cycles globally. Further, Running^110^ hypothesized that global NPP may be treated as carbon cycle planetary boundary, since the change in NPP over past 30 + years has been very limited. Abdi *et al*.^111^ demonstrated that how regional food security study can be studied with MOD 17 data. Tallis *et al*.^112^ found that MOD17 NPP data can be used to calculate the carbon service. Similarly, Mora *et al*.^113^ estimated the growing season potential with MOD 17 NPP data and investigated the possible implications to global food security.

To give a brief idea about the relationship of NPP with climatic variables we evaluated the correlation estimates for precipitation, temperature and soil moisture content with NPP in different time scales. Significant correlation was observed between terrestrial ecosystem productivity and climate variables (see Figure S8 in the supplementary information). It was found that NPP, in general, is positively and strongly correlated with the soil moisture content irrespective of seasons (Figure S9). The correlation of precipitation with NPP was strongly positive in the non-monsoon season in most parts of the country except the barren area and precipitation rich southern-most and north-eastern India. Similarly, the Pearson correlation values obtained from the analysis of NPP and temperature show that increasing temperature favours the productivity only in the north-eastern and northernmost parts of the country. Apart from these two regions, the correlation all over the country was negative indicating low temperature is suitable for better productivity of the terrestrial ecosystems. The correlation analysis explains that the association of NPP of with climate data is significant and efficiently represents overall characteristics of eco-climatic interactions in the country.

### Copula model for evaluating the likelihood of severe damage to ecosystem productivity

Copula-based techniques are advantageous as they offer a significant amount of flexibility in modeling the dependence structure between random variables^114,115^. Also, the existing complexity in dependence structure can be modeled with the help of numerous copula families and their parameters. The Sklar’s theorem^116^ states that a multivariate joint distribution $F({x}_{1},{x}_{2}\ldots {x}_{n})$ can be expressed by a copula as:

1 $$F({x}_{1},{x}_{2},\ldots .{x}_{n})=C[{F}_{{X}_{1}}({x}_{1}),{F}_{{X}_{2}}({x}_{2})\ldots {F}_{{X}_{n}}({x}_{n})]=C({u}_{1},{u}_{2}\ldots {u}_{n})$$

Here, *F*_*Xi*_(*x*_*i*_), is denoted by *u*_*i*_ in the above equation which represents the *i*^*th*^ variable, and *C* is any copula distribution function. Among several available copula families, Archimedean copula (Clayton, Frank and Gumbel) are popularly utilized to model datasets with inconsistencies in their dependence^117–119^. We selected Frank copula type from the Archimedean copula families. Gaussian copulas from the Elliptical families, which carry characteristics of the multivariate Gaussian distributions were also selected for analysis. To add more versatility in the investigation, we have additionally selected Placket copula types. The selection of previously mentioned copula families is advantageous as the density of these copula types allow modeling of wide range of marginals^40^. The parameters for the selected copula functions which represent the association between random variables is also singular which facilitates the understanding of the dependence structure between them^119^. However, prior to modeling the joint dependence, it is important to find the best fit marginal distribution^120^. Five different probability distributions (Gaussian, Weibull, Gamma, Lognormal and Generalized Extreme Value) were compared on the basis of Kolmogorov-Smirnov test statistics^121^. Best copula parameters and corresponding copula function were estimated by Akaike Information Criterion (AIC) and Bayesian Information Criterion (BIC) values, which are efficient indices for relative comparison of models^122^.

Assuming that the net primary productivity (NPP) can be affected by extreme changes in climate, i.e., precipitation, temperature and soil moisture content values, the likelihood of severe reduction in NPP conditioned on an extreme change in climatic conditions was estimated for all grid points at different seasonal and annual scale. Mathematically, the likelihood of a random variable *n*_1_ (NPP) conditioned on *n*_2_ (Precipitation, Temperature or Soil.

Moisture Content) can be represented as:

2 $${F}_{{N}_{1}\le {n}_{1}|{N}_{2}\le {n}_{2}}({n}_{1},{n}_{2})=\frac{C({F}_{{n}_{1}}({N}_{1}),{F}_{{n}_{2}}({N}_{2}))}{{F}_{{n}_{2}}({N}_{2})}=\frac{C({u}_{1},{u}_{2})}{{u}_{2}}$$

To quantify the likelihood of severe impact on the terrestrial ecosystem productivity, we set a threshold of NPP value corresponding to 30^th^ percentile $({n}_{NPP}\le 30 \% )$as an indicator of severe deterioration in primary productivity. The conditional probability distribution corresponding to this NPP threshold was estimated in stressed climate scenarios of precipitation, soil moisture content and temperature, respectively. The stressed scenario is defined by selecting the 20^th^ percentile $(n\le 20 \% )$ of precipitation, soil moisture content and temperature. The selection of threshold is necessary to investigate and demarcate the temporal and spatial risk related to ecosystem functioning. By considering the lower thresholds of climate variable, we can assess the likelihood of extreme changes in ecosystem functioning triggered by extreme climate conditions. Although the choice of the threshold can be self-defined, we, in this study have considered the percentiles such that the sensitivity of NPP to extremely low soil moisture, precipitation and temperature can be captured. To achieve this, we tried the thresholds of 10 to 50 percentiles for soil moisture, precipitation and temperature. It was found that if we eased the climatic constraints, i.e., by selecting greater percentiles of climate data, the conditional probabilities were reduced depicting lower likelihood of drastic reduction in NPP (Figure S4-S7 in the supplementary information). Similarly, to evaluate the likelihood of extreme damage to NPP, first, a threshold as low as 10^th^ percentile was selected. The results showed that likelihood of NPP ≤10% was very low and most parts of the country were safe under stressed climate. The likelihood estimates suggested that even if the climatic condition were highly constrained (say, 10–20 percentiles) the bivariate models showed very minimum chances of NPP ≤10% (Figure S4, S6). This means that NPP ≤10 is way too unrealistic even for highly stressed climatic condition hence, cannot be a suitable threshold for analysis. Similar results were observed for NPP ≤20% threshold resulting in almost moderate or safe likelihood values for all thresholds of precipitation, temperature and soil moisture (Figure S5 and S7). Therefore, it can be concluded that neither an ‘eased’ climatic condition nor too stressed NPP is suitable for analysing the likelihood of extreme changes in NPP. Therefore, a threshold of NPP ≤30% (not too stressed) and soil moisture, precipitation and temperature ≤20% (not too eased) were selected.

### Resilience to extreme conditions

Availability of water is crucial to the occurrence and distribution of NPP^3^. During a dry event, a persistent deficit of soil moisture condition limits the growth of biomass. One of the popular indicators of a dry condition is the Standard Precipitation Index which has been widely utilized in drought-related studies^9,123^. We found the driest month throughout 2001–2010 and observed its impact on primary productivity. It is assumed that a more resilient ecosystem will recover more quickly from a dry condition and attain its temporal mean state. There are several indices available to estimate resilience metrics^124,125^ however, in this study, we have calculated resilience based on the following equation:

3 $${R}_{e}=\frac{NP{P}_{d}}{NP{P}_{m}}$$

Here, the ecosystem resilience indicator R_e_ compares the ecosystem productivity in the driest period (NPP_d_) to its temporal mean value (NPP_m_). Hence, it is understandable that R_e_ should be greater than or equal to 1 for the resilient ecosystems and approach to 0 for the least resilient ecosystems. In other words, the points which show R_e_ value less than or equal to one indicate a non-resilient terrestrial ecosystem region, and for a resilient ecosystem the R_e_ value is greater than one.

## Supplementary information

Revised Supplementary Information

## Data Availability

The datasets generated during and/or analysed during the current study are available from the corresponding author on reasonable request.
