# Supplementary material for: Assessment of Risk and Resilience of Terrestrial Ecosystem Productivity under the Influence of Extreme Climatic Conditions over India
Source: Sci Rep. 2019 Dec 12;9:18923. doi: 10.1038/s41598-019-55067-0 (PMC6908652; doi:10.1038/s41598-019-55067-0)
Supplement: Supplementary file 1 — Revised Supplementary Information [file 41598_2019_55067_MOESM1_ESM.docx]

**Supplementary information for**

**Assessment of Risk and Resilience of Terrestrial Ecosystem Productivity under the Influence of Extreme Climatic Conditions over India**

Srinidhi Jha^1^, Jew Das^2^ and Manish Kumar Goyal^3*^

^1,2, and 3^Discipline of Civil Engineering, Indian Institute of Technology, Indore-453552, India

(*Corresponding author: [vipmkgoyal@gmail.com](mailto:vipmkgoyal@gmail.com) )

**Figure Captions**

**(Added figures have been highlighted in yellow)**

**Figure S1.** River basins IDs and details of Land Cover types considered for analysis.

**Figure S2.** Non-resilient (R_e_<1) area of different river basins at annual scale, and in monsoon and non-monsoon seasons.

**Figure S3.** Percentage of non-resilient (R_e_<1) area of different river basins at annual scale, and in monsoon and non-monsoon seasons.

**Figure S4** Conditional likelihood of $n_{NPP} \leq20\%$ in different scenarios of S/P/T in non-monsoon season to understand the best possible threshold of NPP and climatic data.

**Figure S5** Conditional likelihood of $n_{NPP} \leq20\%$ in different scenarios of S/P/T in non-monsoon season to understand the best possible threshold of NPP and climatic data

**Figure S6** Conditional likelihood of $n_{NPP} \leq20\%$ in different scenarios of S/P/T in monsoon season to understand the best possible threshold of NPP and climatic data.

**Figure S7** Conditional likelihood of $n_{NPP} \leq20\%$ in different scenarios of S/P/T in monsoon season to understand the best possible threshold of NPP and climatic data.

**Figure S8** Significance of correlation (p value) for the combinations of NPP with precipitation, temperature and soil moisture content in different seasons and annual scale at 95% confidence level.

**Figure S9** Correlation of NPP with precipitation, temperature and soil moisture content in different seasons and annual scale.

**Table Captions**

**Table S1.** Area showing high likelihood of severe damage to ecosystem productivity i.e. likelihood of $(n_{NPP}\leq30\%$) greater than 0.5 in non-monsoon season in stressed climate scenario $(n\leq20\%)$ for different river basins.

**Table S2.** Area showing the high likelihood of severe damage to ecosystem productivity i.e. likelihood of $(n_{NPP}\leq30\%$) greater than 0.5 in monsoon season in stressed climate scenario $(n\leq20\%)$ for different river basins.

**Table S3** Percentage and area of vegetation cover showing high likelihood of severe damage to ecosystem productivity i.e. likelihood of $(n_{NPP}\leq30\%$) greater than 0.5 in different seasons and annual scale in stressed climate scenario$(n\leq20\%)$.

**Table S4** Percent and area of non-resilient (i.e. R_e_ <1) river basins in annual scale, and in monsoon and non-monsoon seasons.

**Table S5** Percent and area of non-resilient (i.e. R_e_ <1) vegetation cover types in annual scale, and in monsoon and non-monsoon seasons


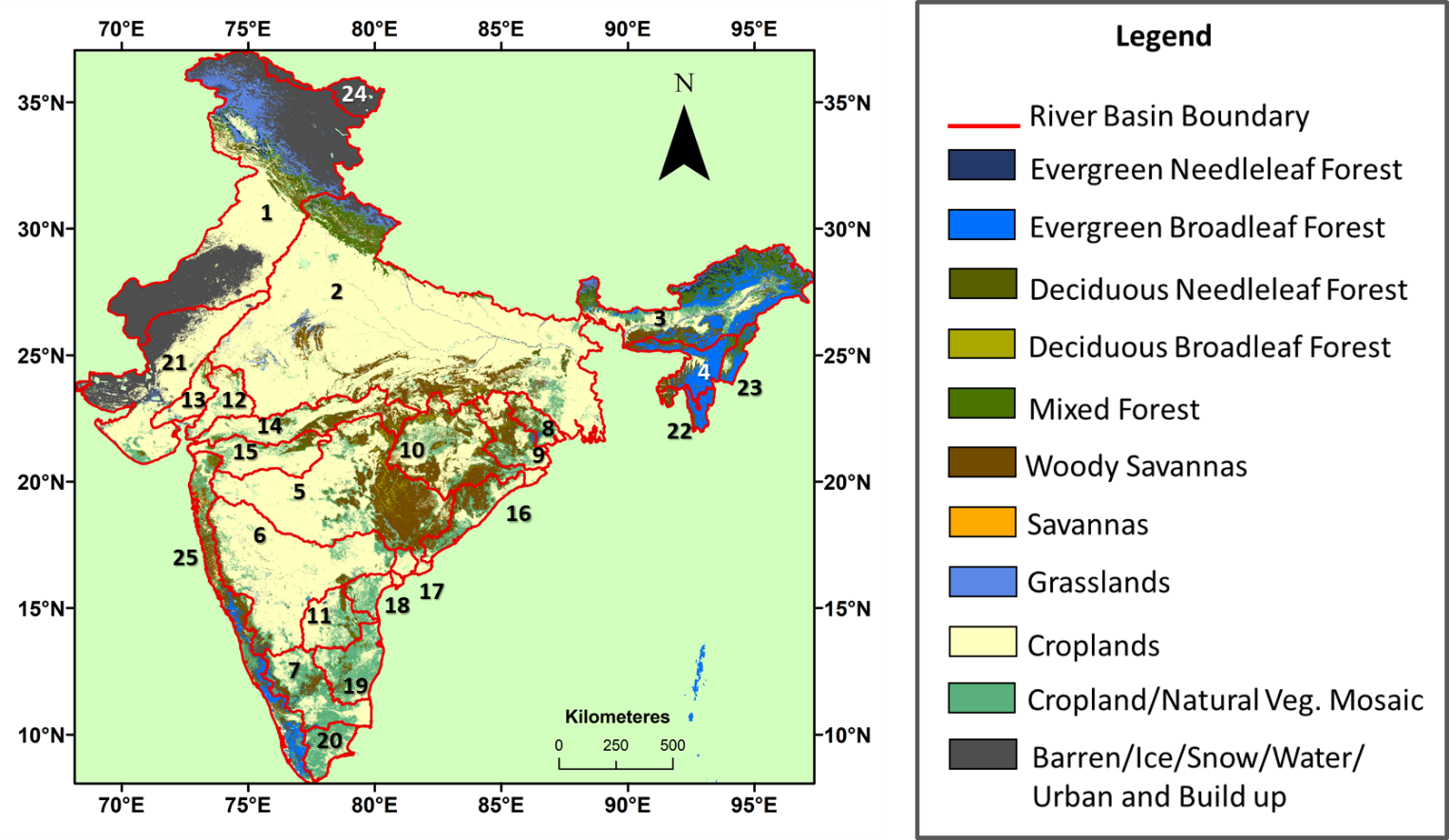


**Figure S1.** River basins IDs and details of Land Cover types considered for analysis (All spatial maps were created using ArcGIS 10.5, www.esri.com/software/ arcgis).


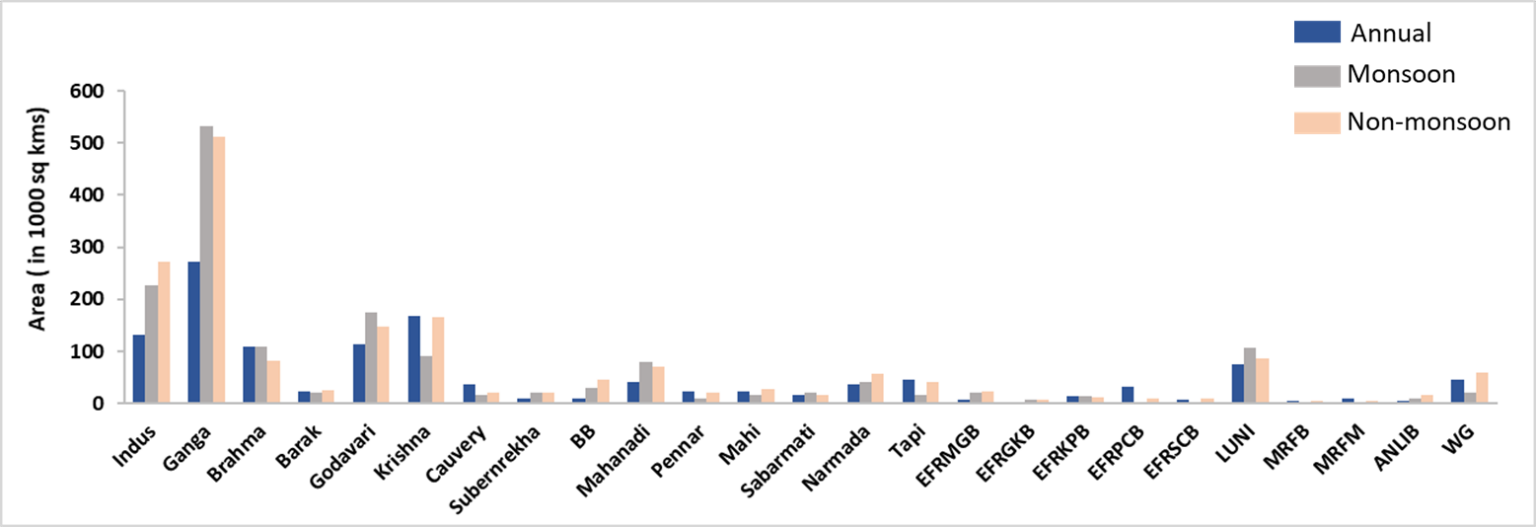


**Figure S2.** Non-resilient (R_e_<1) area of different river basins at annual scale, and in monsoon and non-monsoon seasons.


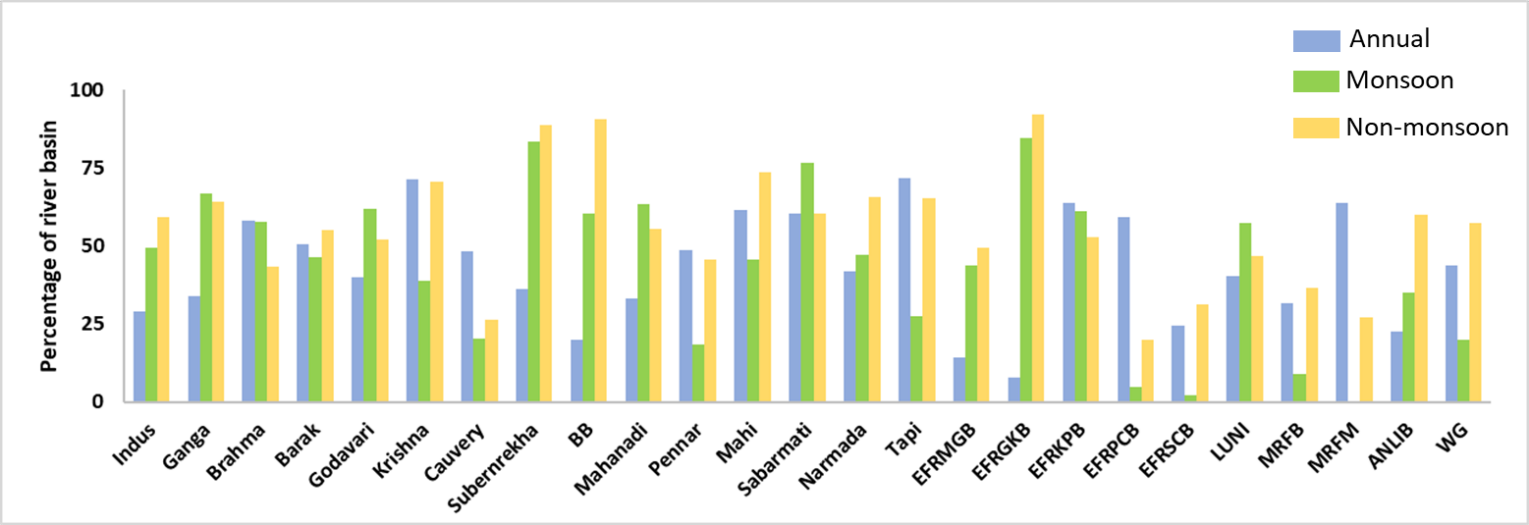


**Figure S3.** Percentage of non-resilient (R_e_<1) area of different river basins at annual scale, and in monsoon and non-monsoon seasons.


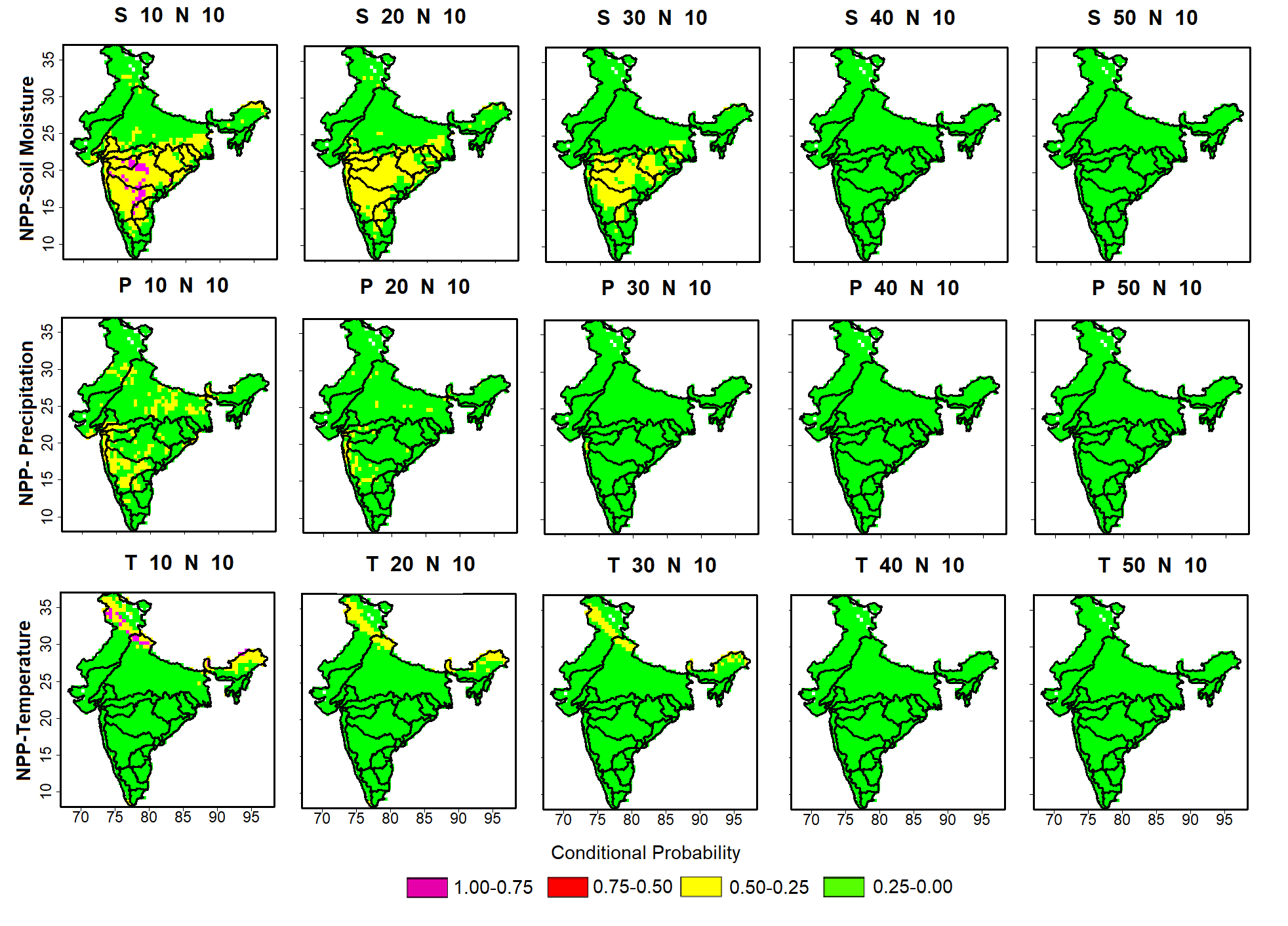


**Figure S4** Conditional likelihood of $n_{NPP} \leq20\%$ in different scenarios of S/P/T in non-monsoon season to understand the best possible threshold of NPP and climatic data.


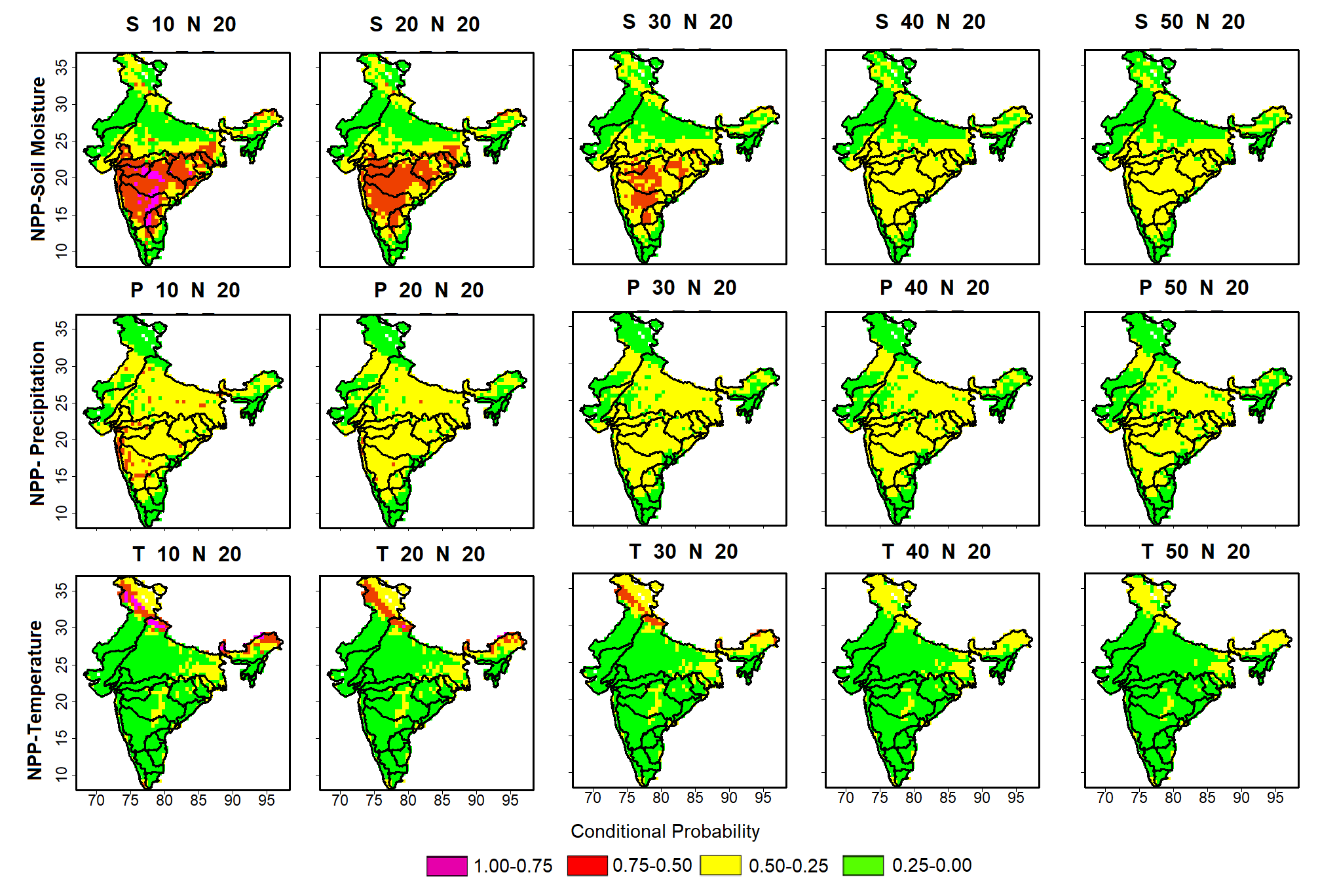


**Figure S5** Conditional likelihood of $n_{NPP} \leq20\%$ in different scenarios of S/P/T in non-monsoon season to understand the best possible threshold of NPP and climatic data.


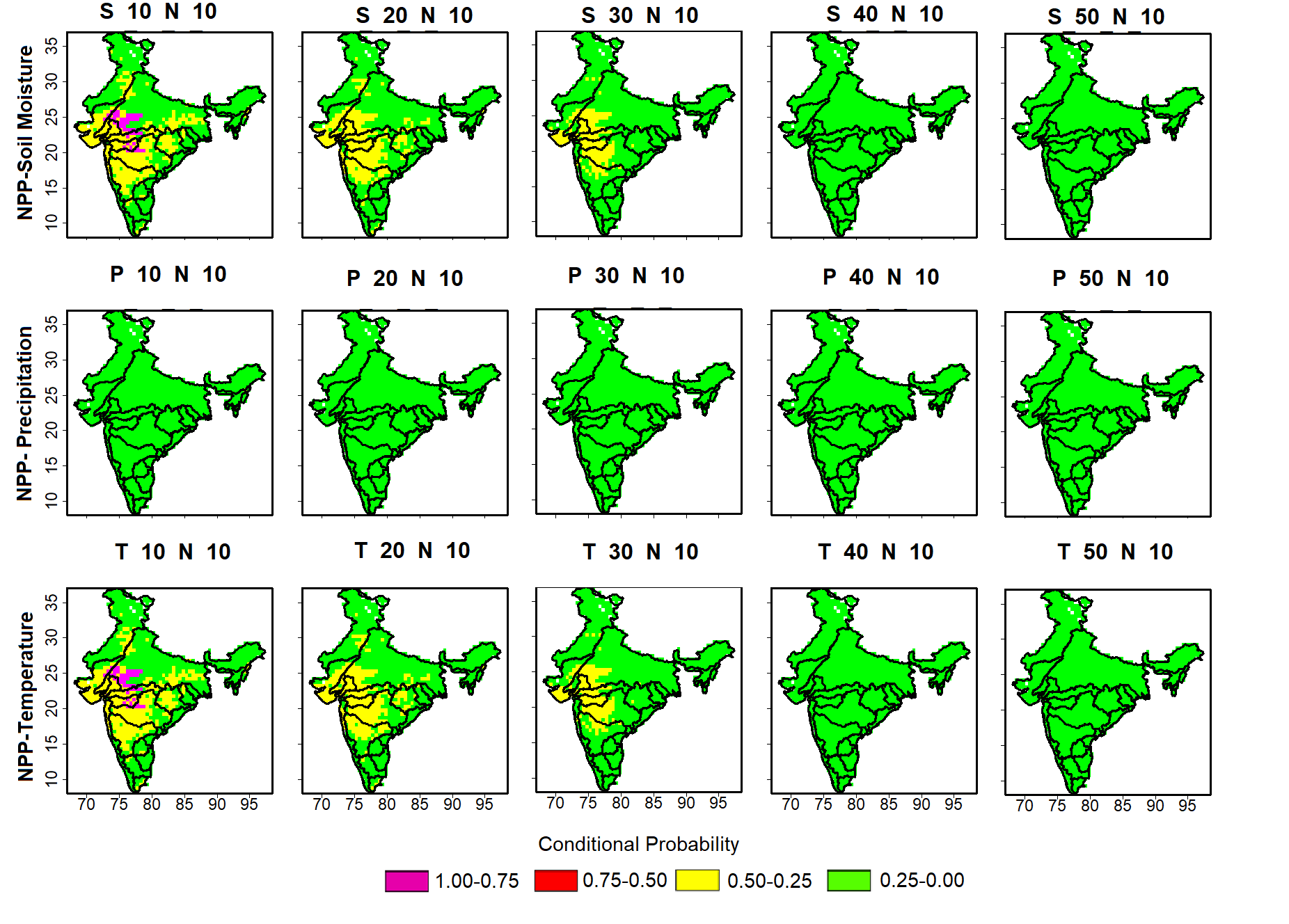


**Figure S6** Conditional likelihood of $n_{NPP} \leq20\%$ in different scenarios of S/P/T in monsoon season to understand the best possible threshold of NPP and climatic data.


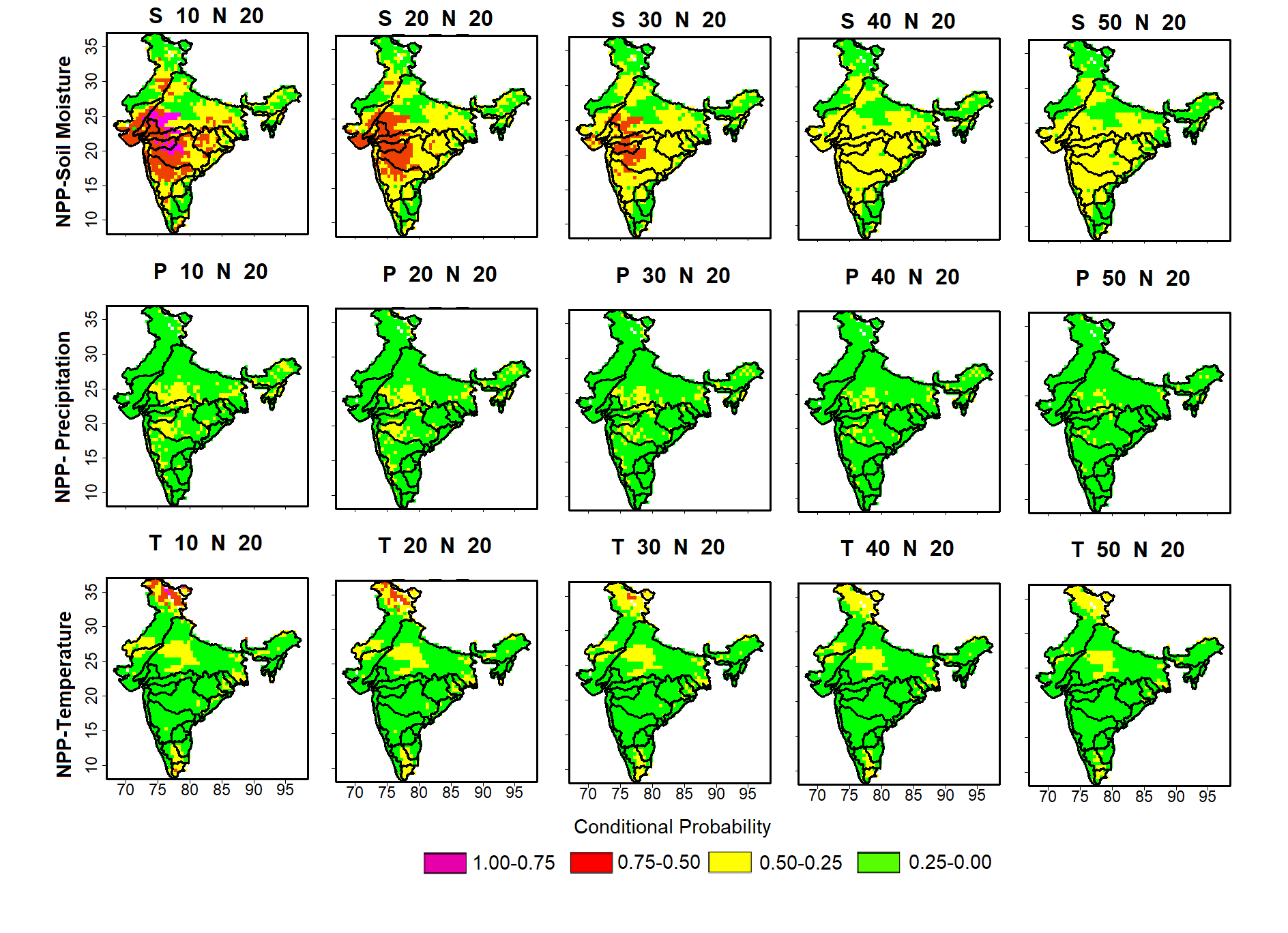


**Figure S7** Conditional likelihood of $n_{NPP} \leq20\%$ in different scenarios of S/P/T in monsoon season to understand the best possible threshold of NPP and climatic data.


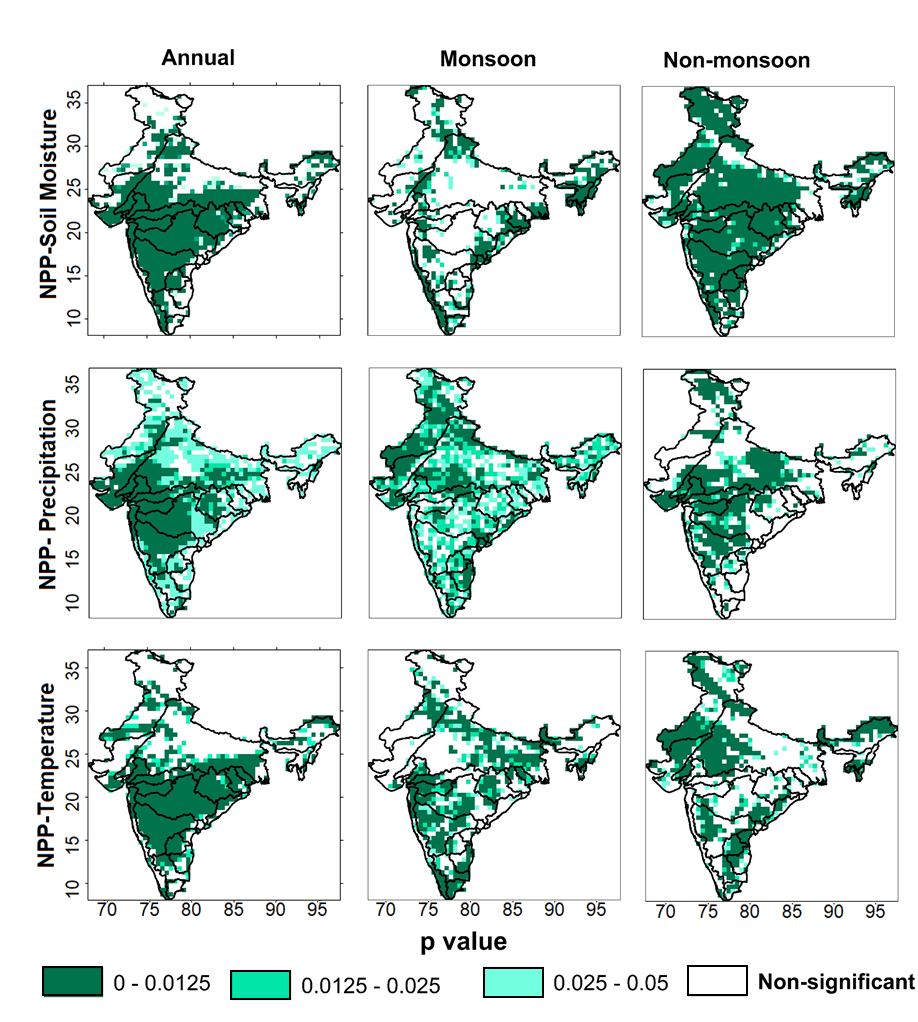


**Figure S8** Significance of correlation (p value) for the combinations of NPP with precipitation, temperature and soil moisture content in different seasons and annual scale at 95% confidence level.


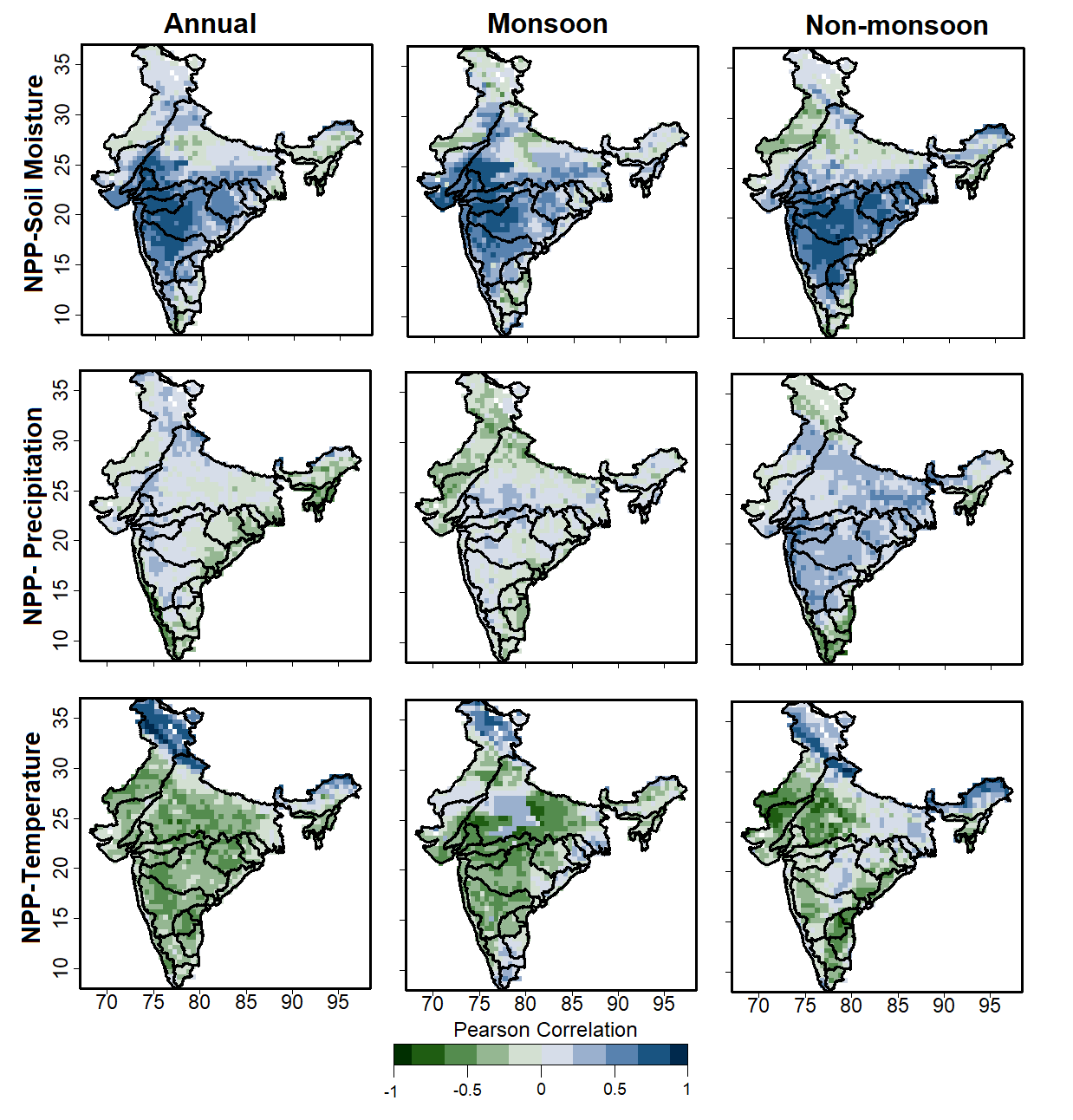


**Figure S9** Correlation of NPP with precipitation, temperature and soil moisture content in different seasons and annual scale.

**Table S1.** Area showing high likelihood of severe damage to ecosystem productivity i.e. $(n_{NPP}\leq30\%$) $\geq0.5$ in non-monsoon season in stressed climate scenario $(n\leq20\%)$ for different river basins.

|  |  | **NPP-Soil Moisture** | | **NPP-Precipitation** | | **NPP-Temperature** | |
| --- | --- | --- | --- | --- | --- | --- | --- |
| **Id** | **Basin** | **Area (%)** | **Area (**$\boldsymbol{k}\boldsymbol{m}^{\boldsymbol{2}}\boldsymbol{)}$ | **Area (%)** | **Area (**$\boldsymbol{k}\boldsymbol{m}^{\boldsymbol{2}}\boldsymbol{)}$ | **Area (%)** | **Area (**$\boldsymbol{k}\boldsymbol{m}^{\boldsymbol{2}}\boldsymbol{)}$ |
| **1** | **Indus** | 4.05 | 18542.18 | 14.45 | 66222.06 | 28.18 | 129133 |
| **2** | **Ganga** | 18.67 | 148999.63 | 27.14 | 216546.1 | 10.04 | 80128.69 |
| **3** | **Brahmaputra** | 25.17 | 47679.88 | 17.83 | 33773.25 | 61.54 | 116550.80 |
| **4** | **Barak** | 5.63 | 2648.88 | 0.00 | 0.00 | 0.00 | 0.00 |
| **5** | **Godavari** | 96.96 | 274821.54 | 33.64 | 95359.76 | 1.40 | 3973.33 |
| **6** | **Krishna** | 92.39 | 217208.35 | 68.17 | 160257.4 | 1.13 | 2648.82 |
| **7** | **Cauveri** | 44.07 | 34435.47 | 21.19 | 16555.51 | 0.00 | 0.00 |
| **8** | **Subarnarekha** | 88.89 | 21191.06 | 44.44 | 10595.53 | 8.33 | 1986.662 |
| **9** | **BB*^1^** | 78.95 | 39733.23 | 23.68 | 11919.97 | 5.26 | 2648.882 |
| **10** | **Mahanadi** | 89.01 | 112577.50 | 16.23 | 20528.84 | 0.52 | 662.06 |
| **11** | **Pennar** | 81.43 | 37746.57 | 42.86 | 19866.62 | 0.00 | 0.00 |
| **12** | **Mahi** | 92.98 | 35097.69 | 43.86 | 16555.51 | 0.00 | 0.00 |
| **13** | **Sabarmati** | 48.84 | 13906.63 | 2.33 | 662.2206 | 0.00 | 0.00 |
| **14** | **Narmada** | 80.92 | 70195.38 | 31.30 | 27151.04 | 0.00 | 0.00 |
| **15** | **Tapi** | 97.89 | 61586.51 | 48.42 | 30462.15 | 0.00 | 0.00 |
| **16** | **EFRMGB*^2^** | 70.42 | 33111.03 | 22.54 | 10595.53 | 0.00 | 0.00 |
| **17** | **EFRGKB*^3^** | 0.00 | 0.00 | 0.00 | 0.00 | 0.00 | 0.00 |
| **18** | **EFRKPB*^4^** | 58.33 | 13906.63 | 8.33 | 1986.662 | 0.00 | 0.00 |
| **19** | **EFRPCB*^5^** | 22.22 | 11919.97 | 0.00 | 0.00 | 0.00 | 0.00 |
| **20** | **EFRSCB*^6^** | 0.00 | 0.00 | 0.00 | 0.00 | 0.00 | 0.00 |
| **21** | **Luni** | 16.85 | 31124.37 | 8.60 | 15893.29 | 0.00 | 0.00 |
| **22** | **MRBB*^7^** | 0.00 | 0.00 | 4.54 | 652.19 | 4.55 | 662.22 |
| **23** | **MRMB^*8^** | 0.00 | 0.00 | 0.00 | 0.00 | 0.00 | 0.00 |
| **24** | **ANLIB^*9^** | 0.00 | 0.00 | 0.00 | 0.00 | 7.50 | 1986.62 |
| **25** | **WG^*10^** | 30.97 | 31786.59 | 26.45 | 27151.04 | 7.10 | 7284.426 |

*1 Brahmani and Baitarni Basin, *2 East flowing rivers between Mahanadi and Godavari Basin, *3 East flowing rivers between Godavari and Krishna Basin, *3 East flowing rivers between Krishna and Pennar Basin, *4 East flowing rivers between Pennar and Cauvery Basin, *4 East flowing rivers South of Cauvery Basin, *7 Minor rivers draining into Bangladesh Basin, *8 Minor rivers draining into Myanmar Basin, *9 Area of North Ladakh not draining into Indus Basin, *10 Western Ghats

**Table S2.** Area showing the high likelihood of severe damage to ecosystem productivity i.e. $(n_{NPP}\leq30\%$) $\geq0.5$ in monsoon season in stressed climate scenario $(n\leq20\%)$ for different river basins.

|  |  | **NPP-Soil Moisture** | | **NPP-Precipitation** | | **NPP-Temperature** | |
| --- | --- | --- | --- | --- | --- | --- | --- |
| **Id** | **Basin** | **Area (%)** | **Area (**$\boldsymbol{k}\boldsymbol{m}^{\boldsymbol{2}}\boldsymbol{)}$ | **Area (%)** | **Area (**$\boldsymbol{k}\boldsymbol{m}^{\boldsymbol{2}}\boldsymbol{)}$ | **Area (%)** | **Area (**$\boldsymbol{k}\boldsymbol{m}^{\boldsymbol{2}}\boldsymbol{)}$ |
| **1** | **Indus** | 14.02 | 64235.40 | 0.00 | 0.00 | 29.77 | 136417.4 |
| **2** | **Ganga** | 33.20 | 264888.20 | 0.00 | 0.00 | 5.56 | 44368.78 |
| **3** | **Brahmaputra** | 3.50 | 6622.20 | 0.35 | 659.1019 | 1.75 | 3311.103 |
| **4** | **Barak** | 9.86 | 4635.54 | 5.63 | 2648.882 | 0.00 | 0.00 |
| **5** | **Godavari** | 76.87 | 217870.60 | 0.00 | 0.00 | 0.00 | 0.00 |
| **6** | **Krishna** | 82.54 | 194030.60 | 0.00 | 0.00 | 0.00 | 0.00 |
| **7** | **Cauveri** | 16.95 | 13244.41 | 0.00 | 0.00 | 0.00 | 0.00 |
| **8** | **Subarnarekha** | 19.44 | 4635.544 | 0.00 | 0.00 | 8.33 | 1985.215 |
| **9** | **BB** | 21.05 | 10595.53 | 0.00 | 0.00 | 5.26 | 2648.882 |
| **10** | **Mahanadi** | 63.35 | 80128.69 | 0.00 | 0.00 | 0.00 | 0.00 |
| **11** | **Pennar** | 25.71 | 11919.97 | 0.00 | 0.00 | 0.00 | 0.00 |
| **12** | **Mahi** | 100.00 | 37746.57 | 0.00 | 0.00 | 0.00 | 0.00 |
| **13** | **Sabarmati** | 100.00 | 28475.48 | 0.00 | 0.00 | 0.00 | 0.00 |
| **14** | **Narmada** | 78.63 | 68208.72 | 0.00 | 0.00 | 0.00 | 0.00 |
| **15** | **Tapi** | 98.95 | 62248.73 | 0.00 | 0.00 | 0.00 | 0.00 |
| **16** | **EFRMGB** | 4.23 | 1986.66 | 0.00 | 0.00 | 0.00 | 0.00 |
| **17** | **EFRGKB** | 0.00 | 0.00 | 0.00 | 0.00 | 0.00 | 0.00 |
| **18** | **EFRKPB** | 0.00 | 0.00 | 0.00 | 0.00 | 0.00 | 0.00 |
| **19** | **EFRPCB** | 0.00 | 0.00 | 0.00 | 0.00 | 0.00 | 0.00 |
| **20** | **EFRSCB** | 44.44 | 13244.41 | 0.00 | 0.00 | 17.78 | 5297.765 |
| **21** | **Luni** | 69.18 | 127808.60 | 0.00 | 0.00 | 1.08 | 1986.662 |
| **22** | **MRBB** | 0.00 | 0.00 | 0.00 | 0.00 | 0.00 | 0 |
| **23** | **MRMB** | 36.36 | 5297.76 | 0.00 | 0.00 | 0.00 | 0.00 |
| **24** | **ANLIB** | 0.00 | 0.00 | 0.00 | 0.00 | 60.00 | 0.00 |
| **25** | **WG** | 43.87 | 45031 | 0.00 | 0.00 | 0.65 | 664.224 |

**Table S3** Percentage and area of vegetation cover showing high likelihood of severe damage to ecosystem productivity i.e. $(n_{NPP}\leq30\%)$ $\geq0.5$ in different seasons and annual scale in stressed climate scenario$(n\leq20\%)$.

|  | **NPP-Soil Moisture** | | **NPP-Precipitation** | | **NPP-Temperature** | |
| --- | --- | --- | --- | --- | --- | --- |
| **Vegetation Type** | **Area (%)** | **Area (km^2^)** | **Area (%)** | **Area (km^2^)** | **Area (%)** | **Area (km^2^)** |
| **Annual** | | | | | | |
| **ENF** | 28.57 | 2648.88 | 35.71 | 3311.10 | 57.14 | 5297.76 |
| **EBF** | 3.27 | 3311.10 | 0.65 | 662.22 | 4.58 | 4635.54 |
| **DNF** | 15.63 | 3309.99 | 6.25 | 1324.44 | 28.13 | 5959.99 |
| **DFB** | 35.19 | 12582.19 | 3.70 | 1321.23 | 16.67 | 5958.30 |
| **MF** | 26.24 | 24502.16 | 10.64 | 9933.31 | 42.55 | 39733.23 |
| **WS** | 43.15 | 110590.84 | 1.03 | 2648.88 | 4.39 | 11257.75 |
| **SAV** | 37.08 | 21853.28 | 10.11 | 5959.99 | 13.48 | 7946.65 |
| **GL** | 32.60 | 39071.01 | 13.81 | 16555.51 | 45.30 | 54302.09 |
| **CL** | 49.61 | 760891.45 | 2.89 | 44368.78 | 1.42 | 21853.28 |
| **CNV** | 24.92 | 54964.31 | 3.60 | 7946.65 | 4.50 | 9933.31 |
| **Monsoon** | | | | | | |
| **ENF** | 0.00 | 0.00 | 0.00 | 0.00 | 36.02 | 3339.84 |
| **EBF** | 6.54 | 6622.21 | 0.00 | 0.00 | 0.64 | 662.22 |
| **DNF** | 15.63 | 3311.10 | 3.12 | 659.21 | 9.38 | 1986.59 |
| **DFB** | 14.81 | 5297.76 | 3.52 | 1260.00 | 5.56 | 1982.61 |
| **MF** | 10.64 | 9933.31 | 0.70 | 653.51 | 4.26 | 3973.32 |
| **WS** | 35.14 | 90062.00 | 0.00 | 0.00 | 2.07 | 5297.76 |
| **SAV** | 38.20 | 22515.50 | 0.00 | 0.00 | 6.74 | 3969.32 |
| **GL** | 28.73 | 34435.47 | 0.00 | 0.00 | 27.07 | 32448.81 |
| **CL** | 54.58 | 837046.81 | 0.09 | 1324.44 | 2.55 | 39071.01 |
| **CNV** | 27.33 | 60262.07 | 0.00 | 0.00 | 5.41 | 11919.97 |
| **Non-monsoon** | | | | | | |
| **ENF** | 28.57 | 2648.88 | 28.57 | 2648.88 | 85.71 | 7946.65 |
| **EBF** | 5.23 | 5297.76 | 3.27 | 3311.10 | 18.30 | 18542.18 |
| **DNF** | 21.88 | 4635.54 | 6.25 | 1324.44 | 43.75 | 9271.09 |
| **DFB** | 48.15 | 17217.74 | 7.41 | 2648.88 | 31.48 | 11257.75 |
| **MF** | 29.08 | 27151.04 | 8.51 | 7946.65 | 56.03 | 52315.43 |
| **WS** | 65.89 | 168866.25 | 18.60 | 47679.88 | 6.72 | 17217.74 |
| **SAV** | 38.20 | 22515.50 | 19.10 | 11257.75 | 20.22 | 11919.97 |
| **GL** | 35.36 | 42382.12 | 16.57 | 19866.62 | 46.96 | 56288.75 |
| **CL** | 43.83 | 672153.89 | 36.83 | 564874.15 | 5.01 | 76817.59 |
| **CNV** | 42.94 | 94697.54 | 16.52 | 36422.13 | 10.21 | 22515.50 |

**Table S4** Percent and area of non-resilient (i.e. R_e_ <1) river basins in annual scale, and in monsoon and non-monsoon seasons.

|  |  | **Annual** | | **Monsoon** | | **Non-monsoon** | |
| --- | --- | --- | --- | --- | --- | --- | --- |
|  |  | **Area (%)** | **Area (**$\boldsymbol{k}\boldsymbol{m}^{\boldsymbol{2}}\boldsymbol{)}$ | **Area (%)** | **Area (**$\boldsymbol{k}\boldsymbol{m}^{\boldsymbol{2}}\boldsymbol{)}$ | **Area (%)** | **Area (**$\boldsymbol{k}\boldsymbol{m}^{\boldsymbol{2}}\boldsymbol{)}$ |
| **1** | **Indus** | 28.9 | 132444.1 | 49.28 | 225817.2 | 59.25 | 271510.43 |
| **2** | **Ganga** | 34.02 | 271510.4 | 66.64 | 531763.1 | 64.15 | 511896.50 |
| **3** | **Brahmaputra** | 58.04 | 109928.6 | 57.69 | 109266.4 | 43.36 | 82115.35 |
| **4** | **Barak** | 50.7 | 23839.94 | 46.48 | 21853.28 | 54.93 | 25826.60 |
| **5** | **Godavari** | 39.95 | 113239.7 | 61.92 | 175488.5 | 52.1 | 147675.18 |
| **6** | **Krishna** | 71.55 | 168204 | 38.87 | 91386.44 | 70.7 | 166217.36 |
| **7** | **Cauvery** | 48.31 | 37746.57 | 20.34 | 15893.29 | 26.27 | 20528.83 |
| **8** | **Subernrekha** | 36.11 | 8608.86 | 83.33 | 19866.62 | 88.89 | 21191.05 |
| **9** | **BB** | 19.74 | 9933.30 | 60.53 | 30462.15 | 90.79 | 45693.22 |
| **10** | **Mahanadi** | 32.98 | 41719.9 | 63.35 | 80128.69 | 55.5 | 70195.38 |
| **11** | **Pennar** | 48.57 | 22515.5 | 18.57 | 8608.86 | 45.71 | 21191.05 |
| **12** | **Mahi** | 61.4 | 23177.72 | 45.61 | 17217.74 | 73.68 | 27813.26 |
| **13** | **Sabarmati** | 59.50 | 16970.02 | 76.74 | 21853.28 | 60.47 | 17217.74 |
| **14** | **Narmada** | 41.98 | 36422.13 | 47.33 | 41057.68 | 65.65 | 56950.96 |
| **15** | **Tapi** | 71.58 | 45031 | 27.37 | 17217.74 | 65.26 | 41057.67 |
| **16** | **EFRMGB** | 14.08 | 6622.20 | 43.66 | 20528.84 | 49.3 | 23177.72 |
| **17** | **EFRGKB** | 7.69 | 661.15 | 84.62 | 7284.42 | 92.31 | 7946.65 |
| **18** | **EFRKPB** | 63.89 | 15231.07 | 61.11 | 14568.85 | 52.78 | 12582.19 |
| **19** | **EFRPCB** | 59.26 | 31786.59 | 4.94 | 2648.82 | 19.75 | 10595.52 |
| **20** | **EFRSCB** | 24.44 | 7284.426 | 2.22 | 662.26 | 31.11 | 9271.08 |
| **21** | **LUNI** | 40.5 | 74830.93 | 57.35 | 105955.3 | 46.59 | 86088.67 |
| **22** | **MRFB** | 31.82 | 4635.54 | 9.09 | 1324.41 | 36.36 | 5297.76 |
| **23** | **MRFM** | 63.64 | 9271.08 | 0.00 | 0.00 | 27.27 | 3973.32 |
| **24** | **ANLIB** | 22.5 | 5959.98 | 35 | 9271.08 | 60 | 15893.29 |
| **25** | **WG** | 43.87 | 45031 | 20 | 20528.84 | 57.42 | 58937.63 |

**Table S5** Percent and area of non-resilient (i.e. R_e_ <1) vegetation cover types in annual scale, and in monsoon and non-monsoon seasons.

|  | **Annual** | | **Monsoon** | | **Non-monsoon** | |
| --- | --- | --- | --- | --- | --- | --- |
|  | **Area (%)** | **Area (**$\boldsymbol{k}\boldsymbol{m}^{\boldsymbol{2}}\boldsymbol{)}$ | **Area (%)** | **Area (**$\boldsymbol{k}\boldsymbol{m}^{\boldsymbol{2}}\boldsymbol{)}$ | **Area (%)** | **Area (**$\boldsymbol{k}\boldsymbol{m}^{\boldsymbol{2}}\boldsymbol{)}$ |
| **ENF** | 64.29 | 5959.99 | 42.86 | 3973.32 | 35.71 | 3311.10 |
| **EBF** | 52.94 | 53639.87 | 28.76 | 29137.71 | 37.25 | 37746.57 |
| **DNF** | 56.25 | 11919.97 | 40.63 | 8608.87 | 34.38 | 7284.43 |
| **DFB** | 51.85 | 18542.18 | 48.15 | 17217.74 | 40.74 | 14568.85 |
| **MF** | 41.13 | 38408.79 | 41.13 | 38408.79 | 47.52 | 44368.78 |
| **WS** | 35.92 | 92048.66 | 51.94 | 133106.34 | 56.33 | 144364.09 |
| **SAV** | 38.20 | 22515.50 | 49.44 | 29137.71 | 56.18 | 33111.03 |
| **GL** | 50.83 | 60924.29 | 48.62 | 58275.41 | 59.12 | 70857.60 |
| **CL** | 43.31 | 664207.24 | 57.21 | 877442.27 | 61.31 | 940353.22 |
| **CNV** | 40.24 | 88737.56 | 42.34 | 93373.10 | 47.15 | 103968.63 |
